# Scaling Evidence-Based Interventions to Improve the Cardiovascular Health of People With Serious Mental Illness

**DOI:** 10.3389/fpsyt.2022.793146

**Published:** 2022-02-04

**Authors:** Christina T. Yuan, Emma E. McGinty, Arlene Dalcin, Stacy Goldsholl, Faith Dickerson, Kimberly A. Gudzune, Gerald J. Jerome, David A. Thompson, Karly A. Murphy, Eva Minahan, Gail L. Daumit

**Affiliations:** ^1^Department of Health Policy and Management, Johns Hopkins University Bloomberg School of Public Health, Baltimore, MD, United States; ^2^Division of General Internal Medicine, Johns Hopkins School of Medicine, Baltimore, MD, United States; ^3^Sheppard Pratt, Baltimore, MD, United States; ^4^Department of Kinesiology, Towson University, Towson, MD, United States; ^5^Department of Anesthesiology and Critical Care Medicine, Johns Hopkins School of Medicine, Baltimore, MD, United States

**Keywords:** implementation strategies, scale-up, serious mental illness, cardiovascular health, evidence-based interventions, implementation

## Abstract

People with serious mental illnesses (SMIs) experience excess mortality, driven in large part by high rates of cardiovascular disease (CVD), with all cardiovascular disease risk factors elevated. Interventions designed to improve the cardiovascular health of people with SMI have been shown to lead to clinically significant improvements in clinical trials; however, the uptake of these interventions into real-life clinical settings remains limited. Implementation strategies, which constitute the “how to” component of changing healthcare practice, are critical to supporting the scale-up of evidence-based interventions that can improve the cardiovascular health of people with SMI. And yet, implementation strategies are often poorly described and rarely justified theoretically in the literature, limiting the ability of researchers and practitioners to tease apart why, what, how, and when implementation strategies lead to improvement. In this Perspective, we describe the implementation strategies that the Johns Hopkins ALACRITY Center for Health and Longevity in Mental Illness is using to scale-up three evidenced-based interventions related to: (1) weight loss; (2) tobacco smoking cessation treatment; and (3) hypertension, dyslipidemia, and diabetes care for people with SMI. Building on concepts from the literature on complex health interventions, we focus on considerations related to the *core function* of an intervention (i.e., or basic purposes of the change process that the health intervention seeks to facilitate) vs. the *form* (i.e., implementation strategies or specific activities taken to carry out core functions that are customized to local contexts). By clearly delineating how implementation strategies are operationalized to support the interventions' core functions across these three studies, we aim to build and improve the future evidence base of how to adapt, implement, and evaluate interventions to improve the cardiovascular health of people with SMI.

## Introduction

Cardiovascular disease (CVD) is the primary cause of preventable death for people with serious mental illnesses (SMIs) (1), due in large part to elevated rates of CVD risk factors (obesity, hypertension, dyslipidemia, and diabetes mellitus) and risk behaviors (tobacco smoking, physical inactivity, and unhealthy diet) that are 1.5–3 times higher in people with SMI than in the overall population (2–5). These disproportionately high rates are driven by a number of factors. At the patient level, metabolic side effects of psychotropic medications (6, 7) and shared pathophysiology between certain CVD and SMI conditions (e.g., altered inflammatory processes) (8, 9) can directly affect cardiovascular health; whereas socioeconomic risks, lack of social support, and cognitive impairment can contribute to suboptimal health habits and impede productive engagement with the healthcare system (10, 11). At the level of care delivery, the poor integration between general medical and specialty mental healthcare likely contributes to significant disparities in the levels of guideline-concordant care delivered to patients with SMI (12, 13). Primary care physicians may lack the comfort or experience in treating people with SMI, mental health specialists may view physical healthcare as outside their purview, and importantly, delivery systems often do not support or reimburse coordination efforts.

Fortunately, there are evidence-based interventions that can help improve the cardiovascular health of people with SMI. A growing number of behavioral interventions tailored to people with SMI have been shown in clinical trials to facilitate weight loss and tobacco smoking cessation (14–16). However, translating the improvements demonstrated in trials has been limited in real-life clinical settings, leading to the well-documented research-to-practice gap that undermines the uptake of many clinical interventions (17). In part, this is because *implementation*—or the process of gaining targeted organizational members' skillful, consistent, and committed use of a practice (18)—is often fraught with challenges, with roughly two-thirds of implementation efforts failing to achieve the intended result (19), and almost half having no effect on outcomes of interest (20). Implementation barriers are numerous and varied, including factors at the provider-level (e.g., providers' lack of self-efficacy to perform an evidence-based practice), organizational-level (e.g., lack of fit between the intervention and current workflows), and system-level (e.g., lack of policies and financing mechanisms that would support sustainable change).

To enhance the uptake of interventions, many translational projects employ *implementation strategies*, which refer to methods or techniques used to improve adoption, implementation, and sustainment of interventions (21). Implementation strategies constitute the “how to” component of changing healthcare practice (21) and range from discrete, relatively “light touch” strategies (e.g., reminder systems for clinicians) to more intensive multi-component strategies that may target multiple levels (e.g., organizational-level, provider-level, and consumer-level). The wide variation in implementation strategies is useful for addressing the diverse array of barriers that may affect implementation outcomes and long-term adoption. However, the tremendous variation, both in terms of the strategies themselves and the ways in which the strategies are described, have led some to characterize the literature on implementation as a ‘Tower of Babel” (22). Although several taxonomies have been developed to organize the types of strategies available (23, 24), implementation strategies are often poorly described in the literature and rarely justified theoretically. Given this lack of precision, implementation scholars are increasingly calling upon the research community to more clearly delineate how implementation strategies are operationalized (21, 25). With more robust specifications of who enacts the strategies and for what purpose, researchers and practitioners will be better able to tease apart why, what, how, and when implementation strategies actually lead to improvement.

In this paper, we describe the implementation strategies being leveraged by the Johns Hopkins ALACRITY Center for Health and Longevity in Mental Illness, a research-practice translation center funded by the National Institute of Mental Health (NIMH) that aims to reduce premature mortality among people with SMI (26). The Center's goals are to develop and test implementation strategies to support the scale-up of three evidenced-based interventions related to: (1) weight loss; (2) tobacco smoking cessation treatment; and (3) hypertension, dyslipidemia, and diabetes care for people with SMI. Building on concepts from the literature on complex health interventions, we first consider the *core functions*, or basic purposes of the change process that the health intervention seeks to facilitate. We next describe the *forms*, or implementation strategies used to carry out the core functions, using Proctor et al.'s recommendations for specifying and reporting implementation strategies (21). By delineating how the selected implementation strategies have been operationalized to support the interventions' core functions across these three studies, we aim to inform and improve future efforts to adapt, implement, and evaluate interventions to improve the cardiovascular health of people with SMI.

### Evidence-Based Interventions to Improve Cardiovascular Health

The Johns Hopkins ALACRITY Center for Health and Longevity in Mental Illness aims to develop and test multi-component implementation strategies to support the scale-up of three evidence-based interventions to reduce cardiovascular risk in SMI. The first project, named ACHIEVE-Dissemination (or ACHIEVE-D for short), is a 6-month evidence-based behavioral weight loss program tailored to adults with SMI that is being delivered in Psychiatric Rehabilitation Programs throughout Maryland. The intervention was adapted from the ACHIEVE randomized-controlled trial (RCT) (15, 27), in which participants who received the intervention (i.e., group and individual weight management sessions and group exercise classes primarily delivered by trained interventionists) experienced clinically significant weight loss (7 lbs. at 18 months). To promote scale-up of the intervention so that it could be implemented by mental health program staff, we adapted the ACHIEVE program using the Enhanced Replicating Effective Programs (REP) Framework (28) to increase the acceptability and feasibility of the ACHIEVE-D curriculum in community settings.

The second project, IMPACT, is a 12-month intervention to support the uptake of evidence-based tobacco smoking cessation treatment (14, 29) in community mental health clinics in Maryland. The overarching premise is that clinics should implement the following evidence-based practices: (1) screening for tobacco use in all patients, (2) assessment of willingness to quit for those who smoke, (3) behavioral counseling for those interested in cutting down or quitting smoking, and (4) pharmacotherapy for those interested in cutting down or quitting smoking. The IMPACT intervention is designed for community mental health organization leaders and providers (e.g., therapists including licensed social workers, counselors, and psychologists; and physician, nurse practitioner, and physician assistant prescribers) to deliver the program to patients who attend community mental health clinics and currently smoke tobacco. To increase mental health providers' uptake of the intervention, we are using Gurses et al.'s interdisciplinary framework of clinicians' compliance with evidence-based guidelines (30) to guide our assessment of baseline characteristics of the clinics, providers, and evidence-based practice as well as relevant mechanisms of change (i.e., providers' knowledge and self-efficacy) to be targeted by implementation efforts.

The third project, RHYTHM, is a 12-month care coordination intervention that aims to equip mental health providers with the ability to coordinate guideline-concordant care for hypertension, dyslipidemia, and diabetes mellitus among people with SMI in the context of Psychiatric Rehabilitation Programs implementing behavioral health homes in Maryland (31–33). Guided by the Translating Evidence into Practice (TRIP) model for large-scale knowledge translation into community settings (34), the study team conducted a comprehensive review of clinical guidelines and scholarly literature to create a bundle of evidence-based care processes for hypertension, dyslipidemia, and diabetes tailored for people with SMI. The bundle includes two overarching components: (1) clinical care processes (e.g., annual dilated eye exam for patients with diabetes), and (2) care coordination and management processes (e.g., using a brief form to facilitate communication between a primary care provider and behavioral health home team at the time of a routinely scheduled visit). These evidence-based practices will be implemented using an adapted version of the Comprehensive Unit Safety Program (CUSP) strategy, which seeks to foster a team-based quality improvement culture and reduce preventable harm (31).

### Form vs. Function

These three evidence-based interventions to improve cardiovascular health for people with SMI can be thought of as *complex health interventions* in which (1) the intervention's multiple components interact in a summative and synergistic fashion; (2) individuals delivering and receiving the intervention exhibit a highly complex set of behaviors; (3) changes are required at multiple levels (e.g., organizational, workforce, or patient); (4) outcomes are numerous and variable; and (5) there is some flexibility in how the intervention is implemented (35). Within the literature on complex health interventions, there is a useful distinction between the *core functions* of an intervention, which speaks to the basic purposes of the change process that the health intervention seeks to facilitate; and its *forms*, which speaks to specific strategies or activities that may be customized to local context and that are needed to carry out the core functions (36). With regards to an intervention's core functions, it is important to note that the “basic purposes of the change process” applies to both the clinical/therapeutic/administrative changes associated with improving health outcomes for patients (e.g., reducing consumption of sugar-sweetened beverages in people with SMI) as well as the implementation-related changes associated with putting the clinical or behavioral intervention into practice (e.g., training providers to increase their knowledge of an intervention). In this Perspective, we focus on the core functions of the implementation-related changes and how these map on to the specific forms, or implementation strategies, designed to facilitate these changes.

### Core Functions of the Interventions

All three of the Center's projects share certain *core functions* related to putting the clinical interventions into practice (Table 1). For example, a core function that supports the delivery of guideline-concordant care across all three projects is to create written processes and defined standards including manuals for delivering care. Moreover, since all of the interventions are being implemented by mental health program staff—not research staff—another core function is to educate clinicians and staff to be able to deliver the interventions' components with fidelity and to use skills in motivational interviewing (i.e., an evidence-based and patient-centered communication method) to more effectively engage with clients in conversations around the targeted behaviors. To complement this educational component, a third core function is to provide opportunities to practice the motivational interviewing techniques that mental health program staff are introduced to through training. All of the projects also aim to facilitate a supportive implementation climate at the organizational-level in which mental health program staff perceive that the adoption, implementation, and use of an innovation is expected, rewarded, and supported by the organization (37).

**Table 1:** Overview of the interventions' core functions and forms.

**Intervention**	**Core Functions**	**Forms (implementation strategies)**
All ALACRITY center projects	Create written processes, defined standards, and manuals for delivering guideline-concordant care	Protocol
	Educate clinicians and staff to deliver the intervention	Training
	Provide opportunity to practice motivational interviewing skills when discussing the targeted behavior	Avatar practice modules
	Facilitate a supportive implementation climate for change	Organizational strategy meetings, adapted comprehensive unit safety program (CUSP)
**Setting-specific**		
ACHIEVE-D: 6-month tailored behavioral weight loss intervention delivered by psychiatric rehabilitation program (PRP) staff	Provide tailored feedback to staff on their delivery of the intervention (in the enhanced arm)	Performance coaching
IMPACT: 12-month evidence-based tobacco smoking cessation treatment delivered by community mental health clinic prescribers and therapists	Provide clinical consultation and support	Coaching
		Expert consultation
RHYTHM: 12-month care coordination intervention for hypertension, dyslipidemia, and diabetes mellitus delivered by behavioral health home providers and PRP staff	Foster a team-based quality improvement culture	Adapted comprehensive unit safety program (CUSP)

Several of the core functions are also specific to the design of a particular intervention. In ACHIEVE-D, for example, mental health program staff serve as “coaches” to deliver weight management and exercise sessions. Consequently, a core function specific to this intervention that will be tested in the “enhanced condition” of the project is to provide tailored feedback to coaches on their delivery of these sessions. In IMPACT, in which therapists and prescribers are responsible for delivering smoking cessation treatment, a core function is to provide clinical consultation and support related to behavioral counseling for smoking cessation and prescription of pharmacotherapy. Lastly, for RHYTHM, which seeks to improve care coordination processes, one of this project's core functions is to foster a team-based quality improvement culture where providers work together to identify a patient-centered problem and then address barriers to receipt of evidence-based care for that problem.

### Specifying Implementation Strategies

Whereas the core functions help to clarify the basic purposes of the change process, the corresponding forms—or implementation strategies—illustrate the specific activities that are needed to carry out the core functions and that can be customized to a local context.

In order to describe how implementation strategies have been operationalized with enough detail to enable measurement and reproducibility, Proctor et al. (21) recommends that researchers specify the: (1) actor(s), (2) action(s), (3) action target(s), (4) temporality, (5) dose, (6) implementation outcomes affected, and (7) theoretical, empirical, or pragmatic justification. The *actor* is defined as the stakeholder who actually delivers the implementation strategy, which for all three Center projects includes faculty and intervention experts from the Johns Hopkins ALACRITY Center for Health and Longevity in Mental Illness. The *actions* indicate the actions, steps or processes, and sequences of behavior (e.g., provide clinical supervision), whereas *action targets* refer to conceptual targets they attempt to impact (e.g., knowledge about the evidence-based practice). *Temporality* refers to the stage or phase when the strategy is used, *dose* refers to the dosage or intensity of the strategy, and *implementation outcomes* typically refer to Proctor et al.'s (38) taxonomy of implementation outcomes (acceptability, adoption, appropriateness, feasibility, fidelity, implementation cost, penetration, and sustainability). Last, the *justification* includes the rationale for why a strategy is being used.

For the Center's three interventions, the core functions of the changes process that are shared across projects also have corresponding implementation strategies in common, although the strategies vary in terms of how they are being operationalized (Table 2). For example, in order to support the core function of educating clinicians and staff to deliver the intervention, all of the projects include synchronous training that is delivered virtually in real-time as an implementation strategy. However, the dose of the training ranges from 2 h (for prescribers in IMPACT) to 15 h (for coaches in ACHIEVE-D), reflecting both the amount of content that needs to be covered as well as the feasibility of conducting the targeted actions.

**Table 2:** Specification of implementation strategies.

**All ALACRITY center projects**					
**Implementation strategy**	**Action**	**Action targets**	**Temporality**	**Dose**	**Outcome**
Treatment protocol	Provide a manual for delivering the evidence-based practices	Knowledge and self-efficacy of coaches/peer leaders (ACHIEVE-D), therapists/prescribers (IMPACT), and clinic staff (RHYTHM) to deliver the intervention	Ongoing	As needed	Adoption, fidelity
Synchronous online training	Present information needed to implement all intervention components including brief training on motivational interviewing (MI); provide opportunity to practice skills	Knowledge, self-efficacy, and skills of coaches/peer leaders (ACHIEVE-D), therapists/prescribers (IMPACT), and clinic staff (RHYTHM) to deliver the intervention	Pre- implementation	ACHIEVE-D: (15 h) IMPACT: (4 h)/for therapists; (2 h)/for prescribers RHYTHM: (12 h)	Adoption, fidelity
Avatar practice modules	Provide opportunity to practice motivational interviewing techniques for targeted behaviors	Self-efficacy of coaches (ACHIEVE-D), therapists/prescribers (IMPACT), and clinic staff (RHYTHM) in using motivational interviewing techniques	Monthly	ACHIEVE-D: (20 min) IMPACT: (15 min) RHYTHM: (15 min)	Penetration amongst clients, fidelity to the MI method
**Setting specific**						
**ACHIEVE-D**	Performance coaching (for the enhanced arm of the project)	Provide tailored feedback to coaches regarding their delivery of video-taped group sessions	Coaches' ability to deliver group sessions with fidelity to the curriculum; motivational interviewing skill development	Monthly	1 h	Penetration amongst clients, fidelity to the intervention
	Asynchronous online training	Review key concepts and discussion points prior to delivering the module; complete learning activity and quiz for each online training module	Coaches and peer leaders' knowledge, self-efficacy, and skills to deliver upcoming group sessions	Monthly	20 min	Penetration amongst clients Fidelity to the intervention
	Organizational strategy meetings	Provide data feedback on client attendance at group sessions; identify barriers at the individual and organizational levels; support group problem-solving; support learning within teams	Implementation climate and leadership engagement at the organizational level	Monthly	30 min	Adoption, Penetration amongst clients, fidelity to the intervention
**IMPACT**	Asynchronous online training	Present introductory information on core components of IMPACT	Therapists and prescribers' knowledge of the intervention	Pre-implementation	Once (1 h)	Adoption
	Coaching calls	Provide clinical consultation and support for behavioral counseling or pharmacotherapy	Therapists and prescribers' knowledge, skills, and access to expertise	Monthly	(30 min)/therapists (15 min)/prescribers	Fidelity to the intervention
	Expert consultation	Provide support for behavioral counseling or pharmacotherapy	Prescribers and therapists' knowledge and skills, and access to expertise	Ongoing	As needed	Fidelity to the intervention
	Organizational strategy meetings	Provide data feedback on delivery of core components; identify barriers at the organizational level; support group problem-solving	Implementation climate and leadership engagement at the organizational level	Monthly	30 min	Adoption, Penetration amongst clients, Fidelity to the intervention
**RHYTHM**	Adapted comprehen-sive unit safety program (CUSP)	Identify barriers, plan strategies to remove barriers, study and refine strategy; support learning within teams; support team members	Clinic staff members' knowledge, self-efficacy, skills, and access to internal expertise; remove barriers; promote supportive organizational climate for RHYTHM	Monthly	2–3 h	Acceptability, Adoption, Feasibility, Fidelity to the intervention, Penetration amongst clients

***Actors:** All actions performed by faculty and intervention experts from the Johns Hopkins ALACRITY Center for Health and Longevity in Mental Illness*.

***Theoretical justification:** ACHIEVE-D: Enhanced Replicating Effective Programs (REP) Model; IMPACT: Gurses et al.'s interdisciplinary framework of clinicians' compliance with evidence-based guidelines; RHYTHM: Translating Evidence into Practice (TRIP) model*.

To meet the needs of the core functions of the change process that are intervention-specific, the Center is also employing an array of implementation strategies that are particular to each project. For example, to enable a culture of quality improvement at participating sites, the RHYTHM project is leveraging an adapted Comprehensive Unit Safety Program (CUSP) implementation strategy comprised of provider training (which is a discrete strategy common across projects) that is combined with expert facilitation and implementation of a five-step quality improvement process (which are strategies specific to RHYTHM). The CUSP strategy is designed to foster a team-based quality improvement culture, in which clinicians and staff are first trained on the science of quality improvement, and then work in CUSP teams to create a process at their organization for continuously identifying and addressing barriers to evidence-based care. By training providers and putting standard processes to implement evidence-based care in place, the CUSP implementation strategy is designed to improve the organization's culture as well as providers' self-efficacy to deliver guideline-concordant care.

## Discussion

Improving the cardiovascular health of people with SMI is a lynchpin to reducing premature mortality in this population. To effectively scale-up interventions that have demonstrated significant improvements in CVD risks and risk behaviors in clinical trial settings, it is imperative to use implementation strategies that speak to both the core functions of the intervention's change processes *and* the localized implementation context. In this Perspective, we describe how the Johns Hopkins ALACRITY Center for Health and Longevity in Mental Illness is operationalizing implementation strategies to support the core functions of three evidence-based interventions related to weight loss (ACHIEVE-D); tobacco smoking cessation treatment (IMPACT); and hypertension, dyslipidemia, and diabetes mellitus care (RHYTHM) for people with SMI which, in total, address all cardiovascular risk factors. By clearly specifying how the implementation strategies have been operationalized, our goal is to contribute to the evidence base of why, how, and when implementation strategies can lead to supporting successful uptake of interventions, and ultimately, improved patient outcomes.

Moving forward, it will be important to consider how implementation strategies are being adapted to increase their acceptability and feasibility in real-life clinical settings, including modifications to improve their fit with local populations, settings, and contexts. The need to account for such adaptations is particularly salient amidst the backdrop of the COVID-19 pandemic, in which the turbulence of the external environment has prompted the need for significant modifications. For example, for all three of the Center's projects, training for mental health program staff was originally designed to be delivered in-person. With the onset of the pandemic, and ensuing restrictions related to in-person gatherings, the Center's faculty and intervention experts had to quickly pivot to reformat the training for the virtual environment. In order to systematically capture these adaptations, the Center plans to use the FRAME-IS model (39), a comprehensive framework for documenting modifications to implementation strategies, in an effort to specify implementation strategies not just in their intended form, but also in the ways in which they have evolved to respond to localized needs. Moreover, as the effectiveness of implementation strategies will likely vary (40), we plan to measure the fidelity with which the strategies are enacted so that we can better ascertain whether the strategies' effectiveness (or relative lack thereof) can be attributed to the strategy itself or to other contextual factors.

The ALACRITY Center projects are pilot in scope, and are designed to provide a foundation to inform future scale-up of evidence-based interventions to decrease cardiovascular risk in persons with SMI. Future work should incorporate hybrid implemementation effectiveness trials testing how these strategies can support intervention implementation on a larger scale. In addition, the implementation strategies used in the Center's current projects are largely focused on supporting change at the provider- or patient-level. In order to achieve widespread scale-up, an important next step will be to consider how to integrate these strategies with system-level and policy strategies at multiple levels (e.g., governmental, payer, system, and organizational). For example, it will be critical to consider what reimbursement mechanisms or other funding streams would be most appropriate and feasible to incentivize uptake of the evidence-based interventions in community settings. The ALACRITY Center's planned future work will include this policy and system-level focus with the goal to accelerate nationwide scale-up of evidence-based interventions addressing cardiovascular risk, and ultimately, reduce premature mortality in people with SMI.

## Data Availability Statement

The original contributions presented in the study are included in the article/supplementary material, further inquiries can be directed to the corresponding author.

## Author Contributions

CY, EEM, SG, AD, FD, KG, GJ, DT, KM, and GD contributed to the conception and design of the study. CY, EEM, SG, EM, and GD drafted the manuscript and all authors assisted with critically reviewing and revising the manuscript for important intellectual content. EEM and GD were responsible for obtaining funding for the study. All authors contributed to the article and approved the submitted version.

## Funding

The authors gratefully acknowledge support from the National Institute of Mental Health (NIMH) Grant P50MH11584201.

## Conflict of Interest

The authors declare that the research was conducted in the absence of any commercial or financial relationships that could be construed as a potential conflict of interest.

## Publisher's Note

All claims expressed in this article are solely those of the authors and do not necessarily represent those of their affiliated organizations, or those of the publisher, the editors and the reviewers. Any product that may be evaluated in this article, or claim that may be made by its manufacturer, is not guaranteed or endorsed by the publisher.
